# Variation of Efficacy of Filtering Face Pieces Respirators over Time in a Dental Setting: A Pilot Study

**DOI:** 10.3390/dj9040036

**Published:** 2021-03-24

**Authors:** Vittorio Checchi, Marco Montevecchi, Luigi Checchi

**Affiliations:** 1 Unit of Dentistry and Oral-Maxillo-Facial Surgery, Department of Surgery, Medicine, Dentistry and Morphological Sciences, University of Modena and Reggio Emilia, 41125 Modena, Italy; 2 Unit of Dentistry, Department of Biomedical and Neuromotor Sciences, University of Bologna, 40100 Bologna, Italy; m.montevecchi@unibo.it (M.M.); luigi.checchi@unibo.it (L.C.)

**Keywords:** personal protective equipment, mask, respirator, FFP2, aerosol spread, environmental contamination, BFE

## Abstract

Since aerosol continuously persists in dental settings, where different procedures and patients come in succession, the use of oronasal masks is highly recommended. Among them, respirators known as Filtering Face Pieces (FFP) show a protective superiority compared to surgical masks. Even concerning respirators classified as non-reusable, it is not known how many hours of use are necessary to compromise their filtering capacity. The aim of this study is to investigate the variations of filtering capacity of an FFP2 respirator over time, in order to safely optimize the timing of its use. Five respirators were worn by the same operator during clinical activity for different usage times (8, 16, 24, 32, 40 h), and one respirator was kept unused. All respirators underwent a bacterial filtration efficacy (BFE) test. T-test for paired data with Bootstrap technique and Wilcoxon test for paired data compared BFE values of the five tested FFP2s respectively at each time, and the areas with the corresponding values of the control respirator (FFp2-F). A generalized linear mixed effect model (GLM) was applied considering type of respirator and time as fixed effects and intercept as random effect. No significant statistical differences were present in the BFE of each time. Data obtained by the present study highlight the important ability of FFP2s to maintain their BFE over time, suggesting a long lasting protective function.

## 1. Introduction

Aerosol spread is an important issue in operative dentistry. High speed air-driven dental handpieces and ultrasonic scalers produce contaminated spatter and an aerosol that can reach the lungs and, in particular, the terminal bronchioles and non-ciliated alveoli. This aerosol is mainly concentrated within 2 m of the patient and must be considered an important risk for dental professional’s health [1,2].

These aerosol particles have their own movement, partly caused by displacement of air masses and in part by the Brownian motions [3]. An Indian research group showed that the highest aerosol contamination occurs within 60 cm from the patient’s head. It was also calculated that the aerosol cloud generated by the use of an ultrasonic scaler remains suspended in the operating area for at least 30 min after treatment [4]. Moreover, 90% of the bacterial spread emitted by ultrasonic instrumentations has been associated with bacteria that can remain airborne and viable for as long as 24 h [5].

In another study it was found that the air quality in a dental clinic after the treatment of only one patient becomes very bad during the aerosol procedure, despite the presence of a high-speed surgical saliva ejector, and remains mediocre for hours after the procedure [1]. It should also be highlighted that the nebulized particles are made up of 75% microorganisms and viruses, while the rest can consist of dentin, enamel, blood residues, epithelial cells, dentin conditioners, water, saliva, and any other organic and inorganic materials that come in contact with rotating tools [5].

Since the aerosol cloud persists continuously in a dental setting where patients and procedures come in succession and overlap within a day, the use of personal protective equipment is highly recommended, and most of the oro-nasal mask [6]. Among them, respirators known as Filtering Face Pieces (FFP) have a clear protective superiority compared to surgical masks because they are created and specifically tested to obtain the maximum protection in both respiratory directions [6]. Respirators are therefore a mandatory and logical device necessary to protect dental professionals from aerosol inhalation and blood spatter. Italian legislation defines FFPs as, “an equipment...worn by the worker capable of protecting him from health risks...in the performance of his duties” (Art. 74 dlg 81-2008). European legislation provides two different respirators classification standards, and both fall among the standards recognized by the European Committee for Standardization (CEN). According to European standards (EN 149: 2001) FFPs are classified in three distinct subclasses depending on their aerosol filtration efficiency towards powders with granulometry between 0.02 and 2 μ. These subclasses are as follows: FFP1, with total filtering efficiency of at least 80%; FFP2, with a minimum total filtering efficiency of 94% (specifications close to N95 respirators in the USA); and FFP3 with a minimum total filtering efficiency of 99% [7,8]. EN 143: 2007 subdivides respirators in P1/P2/P3 (with an efficiency, respectively, of 80%, 94% and 99.95%) [6]. Besides the protection class, a further and important distinction is the recognition of two classes based on the nature of the aerosol, which are class S for water-based mists and class SL for organic mists. Given the prevailing aqueous base of the dental aerosol, FFP2 class S are recommended [9].

Nonwoven materials are the main material used in aerosols filtration. These can be manufactured from synthetic or natural fibers whereby a web or a coat of the materials are secured together. Melt blown fibers are produced by a hot polymer being blown out of a thin tube which are deposited onto a collector in order to cool and create a web of material [10]. FFP2 masks are made up of multiple materials. Straps are composed of thermoplastic elastomers, nose clips are made of aluminum, nose foam of polyurethane and the filter of polypropylene and the shell and cover web are made of polyester [10]. FFP2 masks consist of multiple layers. Those that are accountable for filtering efficiency are, “polypropylene layers with an embedded electrostatic charge” [11].

Strict requirements and testing have been previously set in place to ensure that these face masks and respirators meet certain standards. There are different methods to measure the filtration efficiency, such as the particulate filtration efficiency (PFE), bacterial filtration efficiency (BFE), and viral filtration efficiency (VFE) [12]. BFE requires aqueous bacterial aerosol droplets (with the diameter size of 3 μm) and measures the capability of the masks to stop the large particles from speech, coughing and sneezing, with the size distribution from 0.6 μm up to thousands of microns expelled by the mask’s wearer [13]. The BFE test offers a number of advantages over other filtration efficiency tests. It has been used with little or no modification for years and provides a standard reference for comparison of filtration materials [6,12]. The mean particle size can be cautiously controlled permitting stage-by-stage analysis. This allows filtration efficiencies to be reported up to >99.9%.

Following European legislation (UNI EN 14683-2019), in order to achieve CE (Conformitè Europeenne) certification, all FFPs for healthcare use are obligated to be resistant to harmful solid and liquid aerosols and to be tested through the BFE test. Even if this test is focused on bacteria (*Staphylococcus aureus*), the filtering ability is based on droplets of about 3 µm in size (3.0 ± 0.3 µm), a dimension which better represents inhaled particles that can reach the lower respiratory system. It has been indicated that a cough typically expels droplets between 5 and 20 microns in size [14]. With SARS-CoV-2 thought to be transmitted through droplets, the BFE test can be considered a proxy for the efficacy of a mask in preventing such transmission.

To date, due to the existing COVID-19 pandemic, a considerable use of respirators is utilized [15,16]. These are often replaced after use, with no exact knowledge of the actual filtering capacity over time, and without any scientific criteria, causing considerable economic and environmental damage. Unlike surgical masks, when properly managed, FFPs can be used for multiple shifts [17]. Until now, however, the time beyond which a respirator must no longer be used has never been studied. Even those that have been classified as non-reusable (NR), to our knowledge it is not known how many hours of continuous use are necessary to jeopardize their filtering capacity.

The aim of this pilot study is to investigate the BFE variation of an FFP2 respirator over time in a clinical periodontal practice, in order to safely optimize the timing of its use.

## 2. Materials and Methods

Six FFP2 respirators (3M Aura FFP2 1862+ NR-D; St. Paul, MN, USA), obtained in 2019 pre-COVID-19, were selected for this study. Nowadays, these respirators present a modification certificate registered in November 2020 (Certificate amendment record BSI review n. 2797:20:3216822). These FFP2 presented an ergonomic, flat-fold, 3-panel design featuring a low breathing resistance filter media, a chin tab and an embossed, fluid resistance, sculpted upper panel to help reduce eyewear fogging. These respirators are recommended and marked as non-reusable (NR), meaning that they can be used for only one single shift (according to EN 149:2001 + A1:2009). Although the manufacturer refused to reveal us the specific composition of the layers of the respirator 3M1862+, the only official indication provided by email from 3 M described four layers made of non-cellulose synthetic fibers based on nonwoven polypropylene spunbond and meltblown.

Each respirator was individually packed in hygienic wrapping to prevent contamination before use. Five of them were worn by the same operator (LC) in a periodontal private practice and were held in place continuously during clinical activity and between each patient. At the end of the day, the respirator was placed into its original wrapping and kept closed into a container. One FFP2 respirator was not opened and was used as a control. Usage times are indicated in Table 1.

FFP2-A was used for 8 h as this length represents the average duration of one working day. No mask was used more than 40 h (which equals five working days), because at that time the mask felt malodorous, and it was incompatible with further clinical activity.

Each FFP2 respirator was worn by the same clinician during his clinical activity, based mainly on non-surgical and surgical periodontal therapy. This included procedures that involve the use of ultrasonic devices and high-speed handpieces, with a consequent generation of a high volume of aerosol and droplets [18]. No air-polishing was ever applied. All clinical procedures were conducted in two operatory rooms with two bottom-hung windows continuously open, and after each working session both windows were kept completely open for at least 15 min. The operator wore a protective shield over his face at all times.

### 2.1. Bacterial Filtration Efficacy Test (BFE)

**Figure 1:**
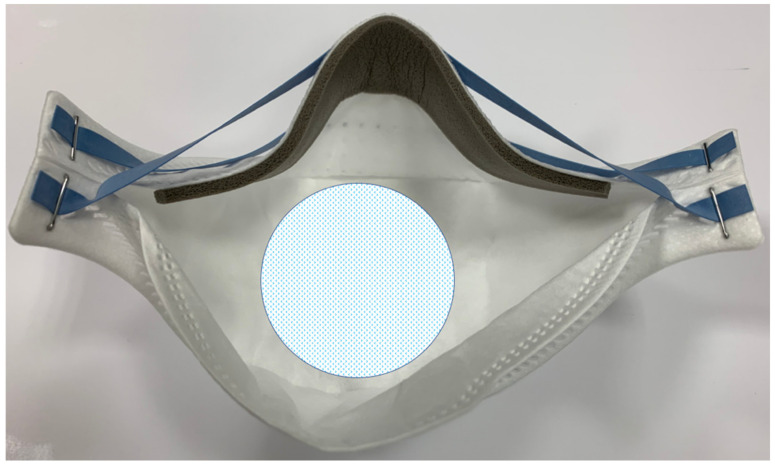
The central area of the internal side of the respirator where the test has been performed. The dimensions of this area are approximately 50 cm^2^ and correspond to the area dimensions of the stages used in the test.

Following the European law UNI EN 14683:2019 + AC:2019, all tested respirators underwent a bacterial filtration efficacy test (BFE). This examination, expressed as a percentage, is indicative of how much part of the mask or respirator represents an effective and efficient barrier for the operator towards a bacterial aerosol. This test is performed on the center of the internal side of the respirator (Figure 1).

**Figure 2:**
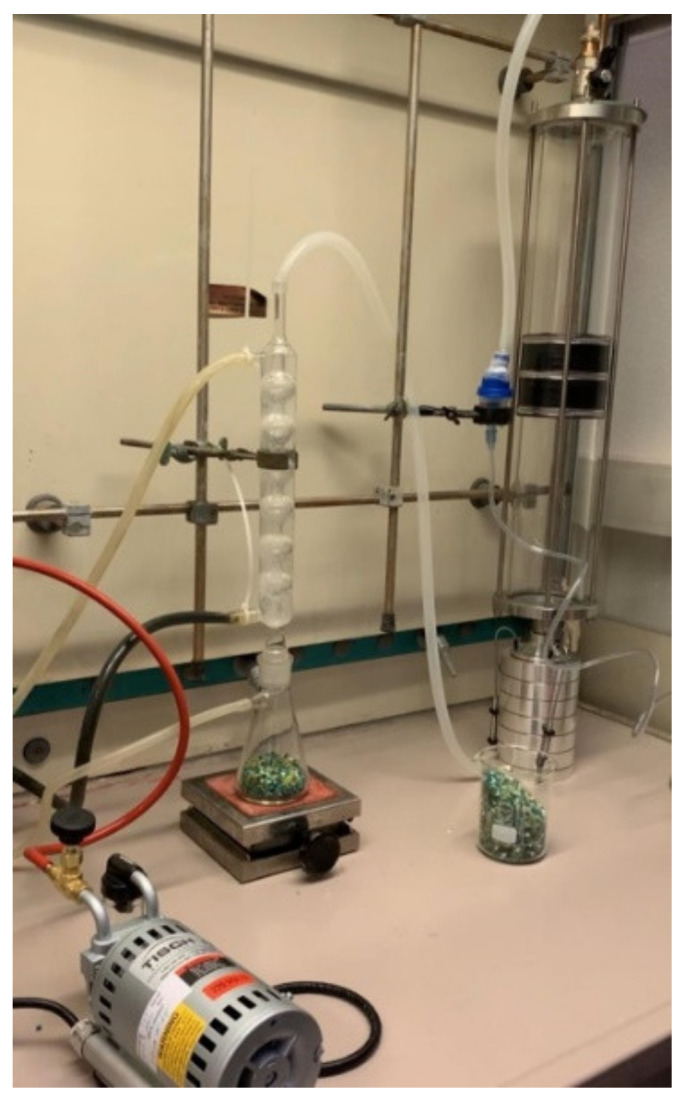
A test bacterial solution made of *Staphylococcus aureus* aerosol is released by a vacuum pump into a cascade impactor.

Initially, the test bacterial solution was inserted into a nebulizer using a disposable pipette and a positive check without any specimen (initial blank) was ran. Triptic Soy Agar (TSA) plates were named with date, stage position and sample number. They were then were placed inside the cascading impactor, one plate on each stage. The test bacterial solution (*Staphylococcus aureus* aerosol) was released by turning on the vacuum pump for a duration of 2 min, adjusting the flow rate based on the speed value continuously measured by the flow meter, so as to have a flow rate of 28.3 L/min (Figure 2).

**Figure 3:**
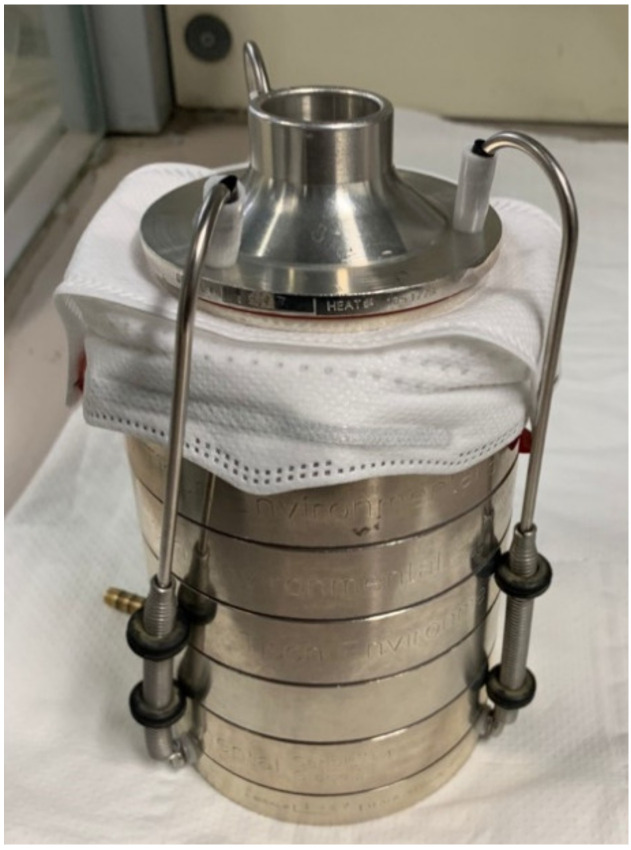
Specimens are fixed in the stand between the first stage and the aerosol inlet cone.

Plates, numbered to indicate their position in the impactor, were then removed from the cascade impactor and stored in the incubator at 37 ± 2 °C from 20 to 52 h. All stages of the impactor were washed and sterilized. Bacterial solution was then reinserted into the nebulizer. Other plates were placed on the respective stages of the impactor, fixing the specimens in the stand between the first stage and the aerosol inlet cone operating the equipment (Figure 3).

All plates were removed from the impactor, washed and sterilized at all stages. These procedures were repeated for all specimens, and when the last sample was tested, an additional positive check was performed (final blank). Finally, all plates were incubated at 37 ± 2 °C for 20–52 h. Each tested area was at least 49 cm^2^.

For each specimen and control, bacterial filtration efficiency B was calculated as a percentage, using the following formula: B = (C − T)/C × 100, where C is the average of the total count on plates for the two positive controls, and T is the total count on plates of the specimen). For each trial and control, the number of bacterial colonies on each plate was calculated to provide the total number of s colony-forming units (UFCs) collected by the cascade impactor. From the positive control plates, the mean aerosol particles size (MPS) of the aerosol of the bacterial test preparation was calculated, using the pre-mentioned formula. The test was conducted in the central area of the internal side of the mask, as imposed by European legislation 14683-2019.

### 2.2. Statistical Analysis

Harmonic mean describes the BFE value because of the decrease of BFE by increasing time. Linear relationship between time and BFE was measured by means of Pearson coefficient. Shapiro–Wilk test was performed aiming to verify the fitting of the raw data to Gaussian distribution. The t- test for paired data with Bootstrap technique compared BFE values of the five tested FFP2s at each time with the corresponding values of the control respirator. The Wilcoxon test for paired data compared BFE values of the tested areas with the corresponding values of the control respirator (FFP2-F). A generalized linear mixed effect model (GLM) was applied considering type of respirator and time as fixed effects, with intercept as a random effect given that BFEs at consecutive times may not be considered independent. α-level was a priori set at 0.05.

## 3. Results

Overall results are shown in Table 1. The BFE harmonic mean is 99.56%. BFE data fitted a Gaussian distribution (Shapiro–Wilk test: *p* = 0–595). No significant statistical differences are present in the BFE of each time between tested respirators and control (*p* = 0.63).

BFE moderately decreases by the increase of the time of usage (Pearson correlation coefficient = −0.67); however, this not statistically significant (*p* > 0.05) relationship was evidenced in less than half of the couples of values (linear determination coefficient = 45%).

GLM confirmed no difference in BFE values between tested respirators and control (*p* = 0.32), not even at different times (interaction respirator-time of usage: *p* = 0.22). Individual results are shown for each respirator in Table 2, Table 3, Table 4, Table 5, Table 6 and Table 7.

BFE areas raw data didn’t fit a Gaussian distribution (Shapiro–Wilk test: *p* = 0.006). No significant statistical differences are present in the BFE between tested respirators and control (Wilcoxon test: *p* = 0.16).

## 4. Discussion

COVID-19 pandemic resulted in a huge increase in demand for Personal Protective Equipment (PPE), three times the number of respirators and six times that of surgical mask [19]. Thus, at the beginning of this pandemic, several countries and health facilities were suffering from in- and out-hospital mass shortage of protective FFP2 respirators [7]. This rise in demand has also resulted in unsuitable PPE being sold that are unfit for use as they do not meet international standards or national legislation [10].

Due to this uncertainty, various disinfection procedures of used respirators have been proposed, not always with satisfactory results. Isopropanol treatment caused significant deterioration of filtration efficacy on three different respirators, whereas ultraviolet germicidal irradiation preserved the filtration efficacy after up to 10 treatments in all the tested respirators [20]. Due to the widespread application of alcohol-based disinfectants, it is important to emphasize that FFP respirators should not be sprayed with alcohol, as it can remove the electrostatic charge from the respirator filter material, severely decreasing the filter’s effectiveness at collecting particles [21]. Microwave-generated steam was shown to be effective in FFP2 decontamination but also incompatible with some kind of respirators, due to arcing observed around some types of metal nose clips and by loss of adhesion of clips to the mask [22]. One-hour treatment in a climate chamber at 70 °C with 75% humidity rate was shown to be adequate for enabling substantial decontamination of FFP2s [7]. Recent literature reviews concluded that the evidence supporting appropriate decontamination strategies for FFP2 respirators remains scarce, although ultraviolet irradiation and vaporized hydrogen peroxide seem to be the current recommended standard [23].

Besides contamination, maintenance and storage are also important factors to be considered when reusing respirators. Pending on specific protocols, the application of good hygiene practices is generally suggested.

Another relevant concern is about the duration of filtration capacity over time. Its deterioration is a crucial aspect that must be clarified in order to safely use FFP2 respirators. In 2004, a literature review focused on respiratory protection against bio-aerosol clearly stressed the need for future research in order to better understand the mechanisms of efficiency degradation, its causes and the exposure indicators that could cause an efficiency reduction [24]. To date, the lack of knowledge about this topic is still not concretely solved.

With regard to non-biologic aerosol, only few publications have investigated this specific aspect. While the filtering efficiency of stored filters remains very stable for years, it has been shown that their performance can decrease when exposed to chemical agents, high humidity, industrial aerosol and temperature [25,26,27].

The mechanism of filter degradation was also investigated by the removal of electrical forces on the filter material, suggesting that filter efficiency is dependent on aerosol loading [28,29]. However, it is still unclear when filters exposed to bio-aerosol undergo similar filter-efficiency degradation. It is important to stress that for bio-aerosol, the number of particles that penetrate through the filter is critical to assess health problems, whereas non-biologic aerosol typically depends on the total mass of particles. This is the reason why the present study is focused on BFE variation.

Our results based on BFE of five respirators measured at 8, 16, 24, 32 and 40 h of usage indicate no significant difference when tested the respirator and control are compared at each time. Moreover, the non-significant effect of time on BFE of the tested respirators is confirmed by multilevel analysis (GLM). In light of these results, it is clear that this type of FFP2 can be considered probably effective for multiple working hours and days. It has to be stressed that its correct fitting must always be verified and any procedure aimed at avoiding direct contamination must be scrupulously followed.

Respiratory protection against bio-aerosol exposure is dependent on several factors and it is mandatory to identify, quantify and categorize the factors that significantly reduce a respirator’s filtering efficiency. Moved by the consciousness of the practical implications of a clinical setting, but aware of the aspects herein discussed, this study is an attempt to standardize, or at least define, the variables potentially involved in this protocol. Certainly, further investigations with a more controlled environmental and non-environmental variables are needed.

The herein used test to evaluate the bacterial filtration efficiency is regulated by the European legislation 14683-2019. It represents the gold standard for surgical masks and respirators. As clearly stated by the specific protocol, it has to be performed on the inner side of the mask and not on the outer side. This technical aspect probably derives by the original proposal to verify the protection of surrounding people instead of the wearer, preventing the transmission of pathogens from health-workers to defied patients or from the surgeon to an open wound. The present study has strictly followed this regulation. In this way, a bidirectional efficiency is not proven, and this could lead to eventual uncertainty for the concrete protection of the wearer. However, it is clearly known, especially during the pandemic, that masks and respirators are concretely able to protect the wearer from droplets and aerosol [6,8,30]. Future investigations on potential BFE variations between inner and outer sides of respirators could be beneficial, although this could non-compliance with the original BFE test protocol.

Specific protocols developed to avoid cross-infections during the present COVID-19 pandemic suggest the use of face shields and double-masking. Regarding the letter action, the herein results support the concept that the additional external mask must be merely considered a barrier for the outer surface of the FFP2. This aspect is very important because if a mask is worn for more than 20 min in an aerosol environment, the outside surface of the mask becomes a nest of pathogenic bacteria [5]. We firmly renew these recommendations and indispensable procedures in order to safely use the tested device.

## 5. Conclusions

Data obtained by the present study highlight the important ability of an FFP2 respirator to maintain its bacterial filtration efficiency over time, and to keep it constant for at least 40 h. Furthermore, the possibility of using an FFP2 NR respirator for several shifts seems acceptable, which means the clinician can make the personal choice of considering FFPs as reusable. Future studies with a higher number of samples, respirators of different brands and with different protection levels and different filtration tests are recommended and are currently ongoing.

## Figures and Tables

**Table 1:** Bacterial filtration efficacy (BFE) values of the six tested FFP2.

FFP2	Time of Usage	BFE Value
FFP2-A	8 h	99.9%
FFP2-B	16 h	99.5%
FFP2-C	24 h	99.7%
FFP2-D	32 h	99.2%
FFP2-E	40 h	99.5%
FFP2-F	Control (no usage)	99.5%

**Table 2:** Individual BFE values of FFP2 A. All samples exceeded the breathability and bacterial filtration tests (BFE). A BFE value greater than or equal to 98% was found on all samples, as required by the UNI EN 14683: 2019 + AC: 2019 standard.

Tested area dimensions	49.8 cm^2^
Sample side exposed to aerosol	Internal
Range	28.3 ± 0.2 L/min
Mean value of the total plate count of the two positive controls	846 CFU
Mean value of the total plate count of the negative control	0 CFU
BFE (%)	99.6

**Table 3:** Individual BFE values of FFP2 B. All samples exceeded the breathability and bacterial filtration tests (BFE). A BFE value greater than or equal to 98% was found on all samples, as required by the UNI EN 14683: 2019 + AC: 2019 standard.

Tested area dimensions	49.4 cm^2^
Sample side exposed to aerosol	Internal
Range	28.3 ± 0.2 L/min
Mean value of the total plate count of the two positive controls	821 CFU
Mean value of the total plate count of the negative control	0 CFU
BFE (%)	99.6

**Table 4:** Individual BFE values of FFP2 C. All samples exceeded the breathability and bacterial filtration tests (BFE). A BFE value greater than or equal to 98% was found on all samples, as required by the UNI EN 14683: 2019 + AC: 2019 standard.

Tested area dimensions	49.5 cm^2^
Sample side exposed to aerosol	Internal
Range	28.3 ± 0.2 L/min
Mean value of the total plate count of the two positive controls	825 CFU
Mean value of the total plate count of the negative control	0 CFU
BFE (%)	99.5

**Table 5:** Individual BFE values of FFP2 D. All samples exceeded the breathability and bacterial filtration tests (BFE). A BFE value greater than or equal to 98% was found on all samples, as required by the UNI EN 14683: 2019 + AC: 2019 standard.

Tested area dimensions	50.2 cm^2^
Sample side exposed to aerosol	Internal
Range	28.3 ± 0.2 L/min
Mean value of the total plate count of the two positive controls	745 CFU
Mean value of the total plate count of the negative control	0 CFU
BFE (%)	99.5

**Table 6:** Individual BFE values of FFP2 E. All samples exceeded the breathability and bacterial filtration tests (BFE). A BFE value greater than or equal to 98% was found on all samples, as required by the UNI EN 14683: 2019 + AC: 2019 standard.

Tested area dimensions	50.4 cm^2^
Sample side exposed to aerosol	Internal
Range	28.3 ± 0.2 L/min
Mean value of the total plate count of the two positive controls	791 CFU
Mean value of the total plate count of the negative control	0 CFU
BFE (%)	99.5

**Table 7:** Individual BFE values of FFP2 F. All samples exceeded the breathability and bacterial filtration tests (BFE). A BFE value greater than or equal to 98% was found on all samples, as required by the UNI EN 14683: 2019 + AC: 2019 standard.

Tested area dimensions	49.9 cm^2^
Sample side exposed to aerosol	Internal
Range	28.3 ± 0.2 L/min
Mean value of the total plate count of the two positive controls	768 CFU
Mean value of the total plate count of the negative control	0 CFU
BFE (%)	99.5

## Data Availability

Not applicable.
